# Synovial biopsy for establishing a definite diagnosis in undifferentiated chronic knee monoarthritis

**DOI:** 10.1186/s12891-023-06138-x

**Published:** 2023-01-11

**Authors:** Soosan G Soroosh, Ali Ghatfan, Abolfazl Farbod, Elahe Meftah

**Affiliations:** 1 Rheumatology Research Center, AJA University of Medical Sciences, Tehran, Iran; 2 501 Hospital, AJA University of Medical Sciences, Tehran, Iran; 3 Headache Department, Iranian Center of Neurological Research, Neuroscience Institute, Tehran University of Medical Sciences, Tehran, Iran; 4 Students’ Scientific Research Center, Tehran University of Medical Sciences, Tehran, Iran

**Keywords:** Knee monoarthritis, Synovial biopsy, monoarthropathy, Chronic synovitis, Undifferentiated arthritis, Monoarticular arthritis

## Abstract

**Background:**

Undifferentiated arthritis is a condition in which the problem cannot be classified into any definite diagnosis category. Various methods have been suggested to clarify the definite diagnosis in this class. The synovial biopsy is suggested as the last diagnostic approach to determine the precise histopathological diagnosis. In this study, we aimed to evaluate the efficacy of synovial biopsy for establishing a definite diagnosis in patients with undifferentiated chronic knee monoarthritis.

**Methods:**

The present retrospective case series was conducted in 2005 in the rheumatology research center of Shariati hospital and the 501 hospital in Tehran, Iran. The study included the synovial biopsy of patients with chronic knee monoarthritis who did not have a definite diagnosis after all the diagnostic steps before the synovial biopsy. Pathology slides of the patients’ synovial biopsy were reevaluated with a senior expert pathologist.

**Results:**

Eighty patients with a mean age of 37.6 ± 17.32 years (range: 6–68) were included, of whom 50% were female. The gap time between the onset of knee monoarthritis and the decision-making for synovial biopsy was 14.34 ± 19.61 months. Histopathologic evaluations revealed non-specific synovitis in 65% of the patients and a definite diagnosis in 35%. The most common definite diagnosis was rheumatoid arthritis (9%), followed by septic arthritis (5%). The most common pathologic findings were endothelial proliferation (89%) and synovial proliferation (88%), and the most common infiltrating cell was lymphocyte (54%). Patients with non-specific synovitis were more likely to have neovascularization, cellular infiltration (*p*-value < 0.001), synovial proliferation, endothelial proliferation (*p*-value = 0.001), pannus formation (*p*-value = 0.009), and fibrosis (*p*-value = 0.022) compared to the patients with a definite pathologic diagnosis. However, age, gender, and the gap time between disease symptoms to synovial biopsy were not significantly different between the different groups of diagnosis (*p*-value > 0.05).

**Conclusion:**

Non-specific synovitis remains the most common histopathologic finding, highlighting the importance of physician expert opinion for most of the patients with undifferentiated chronic knee monoarthritis. Studies with larger samples and immunohistochemistry analyses are needed to clarify this uncategorized entity further.

## Introduction

Musculoskeletal disorders are among the most prevalent reasons for seeking medical help. It is estimated that up to 30% of the population experience at least one musculoskeletal complaint during their lifetime [1, 2]. Among musculoskeletal complaints, disorders with articular origin are prominent. Specifically, knee involvement accounts for one of the most common reasons for chronic pain in the general population [3]. Compared to periarticular lesions, articular involvements tend to last longer than six weeks and become chronic, can affect a diverse number of joints, and cause pain with inflammatory features.

Among the different types of joint involvement, revealing the reason for chronic monoarthritis poses a diagnostic challenge. The etiologies of chronic inflammatory monoarthritis can be classified as indolent infections (e.g., tuberculosis and brucellosis), fungal infections, gout and calcium pyrophosphate crystal deposition disease (CPPD), and immunoinflammatory arthritis. Immunoinflammatory arthritis could be due to autoimmune conditions like spondyloarthritis, systemic lupus erythematosus, or rheumatoid arthritis (RA). Chronic non-inflammatory monoarthritis is attributed to four major groups of osteoarthritis, osteonecrosis, neuropathic joint, and pigmented villonodular synovitis [4, 5]. In addition to the mentioned categories, undifferentiated arthritis refers to the condition that arthritis could not be categorized as rheumatoid arthritis or other classes of definite arthritis. Undifferentiated arthritis might indicate the early phase of an established disease or remain an entity that does not fit into any category of rheumatologic diagnoses [6, 7]. Establishing a definite diagnosis for undifferentiated arthritis disorders is a long-standing challenge in rheumatology. As knee arthritis is one of the most common sites of involvement in undifferentiated monoarthritis [8], exploring the etiologies behind this phenomenon is particularly important.

Evaluation of chronic monoarthritis starts with a thorough history and physical examination and continues with appropriate laboratory and imaging studies. In rare cases, a synovial biopsy is undertaken to achieve a precise diagnosis. Histopathological examination of synovial specimens may be valuable in making an early diagnosis [9]. In addition to the diagnosis, findings of synovial biopsy have prognostic value [6, 10] and alter under response to the treatment [11]. It is reported that, specifically in undifferentiated arthritis, synovial tissue biopsy can facilitate the diagnostic process [6, 12].

As the diagnosis of chronic undifferentiated monoarthritis is problematic, synovial tissue biopsy might offer valuable information as the last diagnostic utility and aid in reaching a precision medicine approach. Synovial biopsy findings are not extensively reviewed in the literature for chronic knee monoarthritis [6, 7]. Thus, we aimed to evaluate synovial tissue biopsy’s clinical implication and efficacy for establishing definite diagnoses in patients previously labeled as having chronic undifferentiated knee monoarthritis.

## Methods

The present retrospective case series was conducted in 2005 in the rheumatology research center of Shariati hospital and the 501 hospital, two tertiary referral teaching hospitals in Tehran, Iran. The study sample included all the patients from the rheumatology clinic of the two mentioned hospitals between 1998 and 2004 with chronic knee monoarthritis who had no definite diagnosis after all the preliminary diagnostic steps and had undergone synovial biopsy as the last diagnostic tool. The preliminary diagnostic tests included complete blood count (CBC), erythrocyte sedimentation rate (ESR), C-Reactive Protein (CRP), uric acid, thyroid stimulating hormone (TSH), glycosylated hemoglobin, urine analysis, antinuclear antibody (ANA), rheumatoid factor, anti-cyclic citrullinated peptides antibodies (APCAs), arthrocentesis, and x-ray radiography of the involved joint. To evaluate indolent infections, we tested the patients with purified protein derivate for tuberculosis, and wright, coombs wright, and 2-mercaptoethanol for brucellosis [13].

Exclusion criteria were prior use of biologic agents, previous intra-articular injection of corticosteroids, active infections, history of synovial biopsy, duration of the symptoms less than six weeks, and a history of surgery in the target joint (excluding arthroscopy). Additionally, patients with samples taken only from the surrounding tissues or with incorrect slide preparation and staining techniques, improper transport and maintenance of the slides, and mislabeled or missing slides were not included.

**Fig. 1:**
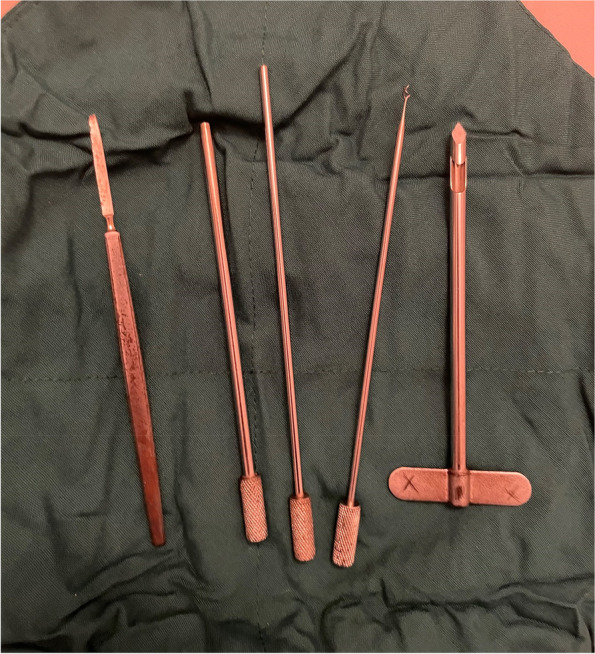
Paul and Beakle needle

An expert rheumatologist performed the biopsy in a closed method with Paul and Beakle needle (Fig. 1). Concomitant synovial fluid and blood sampling were done at the time of the biopsy and were sent for culture and biochemistry analysis. The biopsy for some patients who were referred from the orthopedics ward was taken through an open biopsy technique. An expert pathologist reevaluated all synovial biopsy slides, and the pathologic findings were recorded. Specimens were evaluated regarding synovial proliferation, endothelial proliferation, neovascularization, granuloma, giant cells, pannus formation, fibrosis, hemosiderin deposition, infiltration of inflammatory cells, and any evidence of invasion, dysplasia, or neoplasm. The diagnosis of septic arthritis was confirmed by either observing the evidence of infection in histopathologic examination or positive culture of synovial fluid, synovial tissue, or blood. Where the pathologist could not attribute any definite diagnosis to the examined sample, the sample was labeled as having non-specific synovitis.

A data collection form was designed based on the study parameters. Information was gathered through data collection forms concerning age, gender, duration of symptoms, the involved joint, and histopathologic findings. The relevant data were extracted from the medical and pathology records of the patients.

Statistical analysis was performed with SPSS version 16 for Windows. Descriptive statistics were reported as mean, standard deviation, and range. Frequencies were reported as the total number and percentage. Since the Kolmogorov-Smirnov test confirmed the normal distribution of the sample, Student’s T-test and chi-square test were used for inter-group comparisons. The statistical significance level was considered as *p*-value < 0.05.

## Results

**Fig. 2:**
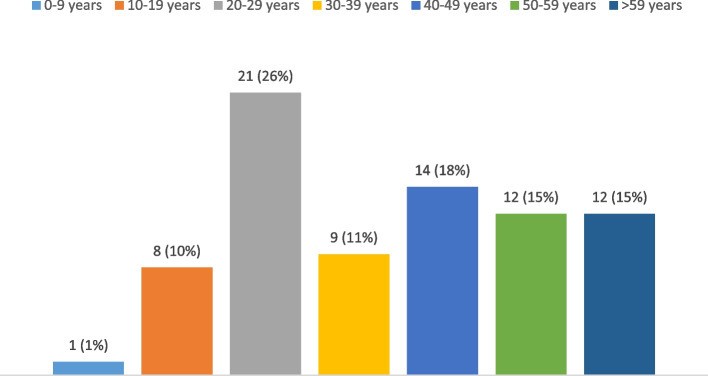
The distribution of age groups in the studied population

One hundred and forty tissue samples were evaluated in the present study, of which 60 were excluded. As a result, 80 patients with chronic knee monoarthritis were eventually included. Gender distribution was precisely equal (1:1). The mean age was 37.6 ± 17.32 years (range: 6–68 years), and the most common age group was 20–29 years (21 patients, 26%, Fig. 2). None of the patients had undergone arthroscopy. The gap between the onset of knee monoarthritis and the decision-making for synovial biopsy was 14.34 ± 19.61 months. The mentioned gap time was zero to six months in 23 (45%), six to twelve months in 15 (29%), and more than twelve months in 13 patients (26%). The right knee was involved in the majority (40 patients, 58%).

The most common final opinion of the pathologist was “non-specific synovitis” in 52 samples (65%). In patients with a definite diagnosis, the most common diagnosis was rheumatoid arthritis in seven (9%), followed by septic arthritis in four (5%). Three (4%) of the diagnoses were malignant. The detailed frequency of the diagnoses is shown in Table 1.

**Table 1:** Definite diagnoses suggested by the pathologist according to the synovial biopsy

**Diagnosis**	**Number of patients (%)**
**Non-specific synovitis**	52 (65)
**Rheumatoid arthritis**	7 (9)
**Septic arthritis**	4 (5)
**Gout**	2 (3)
**Osteoarthritis**	2 (3)
**Pigmented villonodular synovitis**	2 (3)
**Synovial chondromatosis**	2 (3)
**Fibrosis arthritis**	1 (1)
**Giant cell tumor**	1 (1)
**Chondrosarcoma**	1 (1)
**Trauma**	1 (1)
**Metastatic neoplasm**	1 (1)
**Amyloidosis**	1 (1)
**Tuberculosis**	1 (1)
**Juvenile rheumatoid arthritis**	1 (1)

The most common pathologic finding of synovial biopsy specimens was endothelial proliferation in 71 patients (89%), and the most common infiltrating cell was lymphocyte in 43 samples (54%). The details of the pathologic findings are listed in Table 2.

**Table 2:** Histopathologic findings of the pathology samples

**Pathologic findings**	**Number of patients (%)**
**Endothelial proliferation**	71 (89)
**Synovial proliferation**	70 (88)
**Neovascularization**	64 (80)
**Fibrosis**	63 (79)
**Infiltrating cells**	60 (75)
** Lymphocyte**	22 (28)
** Lymphocyte-monocyte**	16 (20)
** Monocyte**	6 (8)
** Lymphocyte-polymorphonuclear**	5 (6)
** Polymorphonuclear**	5 (6)
** Macrophage**	4 (5)
** Monocyte-polymorphonuclear**	2 (3)
**Pannus formation**	52 (65)
**Giant cells**	8 (10)
**Hemosiderin deposition**	8 (10)
**Neoplasm**	5 (6)
**Non-necrotic granulomas**	2 (3)
**Necrotic granulomas**	1 (1)

Overall, 52 patients (65%) were diagnosed with non-specific synovitis, and 28 patients (35%) were diagnosed with a definite histopathologic diagnosis. The “non-specific synovitis” group included 28 females (54%) and 24 males (46%), while the “definite diagnoses” group consisted of 12 females (43%) and 16 males (57%). Statistical analysis did not show a significant difference between the patients with or without a definite diagnosis regarding gender distribution (*p*-value = 0.348). The presence of a definite histopathologic diagnosis was not associated with the gap from the onset of the disease to the decision-making for synovial biopsy (*p*-value = 0.181). The comparison of age and gap duration between disease onset and synovial biopsy did not show any significant difference between the different groups of diagnosis (*p*-values = 0.248 and 0.929, respectively).

Patients with non-specific synovitis were more likely to have neovascularization, cellular infiltration (*p*-value < 0.001), synovial proliferation, endothelial proliferation (*p*-value = 0.001), pannus formation (*p*-value = 0.009), and fibrosis (*p*-value = 0.022) compared to the patients with a definite pathologic diagnosis. The details of the histopathologic findings in each pathologic diagnosis are discussed in Table 3.

**Table 3:** Histopathologic findings in definite diagnoses and non-specific synovitis

**Diagnosis**	**Synovial proliferation**	**Endothelial proliferation**	**Necrotic granuloma**	**Non-necrotic granuloma**	**Giant cells**	**Pannus formation**	**Neovascularization**	**Fibrosis**	**Hemosiderin deposition**	**Infiltrating cells**
Non-specific synovitis	49 (94%)	51 (98%)	0 (0%)	2 (4%)	3 (6%)	37 (71%)	48 (92%)	45 (87%)	4 (8%)	43 (83%)
Crystal disease	1 (50%)	1 (50%)	0 (0%)	0 (0%)	0 (0%)	1 (50%)	1 (50%)	1 (50%)	1 (50%)	1 (50%)
Degenerative disease	4 (80%)	3 (60%)	0 (0%)	0 (0%)	0 (0%)	2 (40%)	3 (60%)	3 (60%)	0 (0%)	1 (20%)
Infectious disease	5 (83%)	4 (67%)	1 (17%)	0 (0%)	0 (0%)	3 (50%)	3 (50%)	4 (67%)	0 (0%)	6 (100%)
Infiltrative disease	0 (0%)	1 (100%)	0 (0%)	0 (0%)	1 (100%)	1 (100%)	1 (100%)	1 (100%)	0 (0%)	1 (100%)
Neoplasm	2 (40%)	2 (40%)	0 (0%)	0 (0%)	4 (80%)	0 (0%)	0 (0%)	1 (20%)	1 (20%)	0 (0%)
Rheumatoid arthritis	8 (100%)	8 (100%)	0 (0%)	0 (0%)	0 (0%)	8 (100%)	8 (100%)	7 (88%)	1 (13%)	7 (78%)
Trauma	1 (100%)	1 (100%)	0 (0%)	0 (0%)	0 (0%)	0 (0%)	0 (0%)	1 (100%)	1 (100%)	1 (100%)

## Discussion

The synovial biopsy is an invasive approach and is not routinely conducted for diagnostic purposes. However, examination of synovial tissue can assist in the diagnosis of some conditions like rheumatoid arthritis (RA), tuberculosis, fungal involvements, and some bacterial infections. The synovial biopsy is considered the final and last choice for establishing the diagnosis of chronic monoarthritis. It is assumed that in cases of undifferentiated arthritis, a synovial biopsy can facilitate the diagnostic process [6, 14]. Results of our study showed that synovial biopsy could establish a definite diagnosis in one-third of cases with undifferentiated chronic knee monoarthritis. However, histopathologic examination revealed nothing but non-specific synovitis in the other two-thirds of the cases. In other studies on chronic monoarthritis, it was observed that the cause of arthritis in 16–62% of cases remains undefined [8, 14, 15].

In the present study, the definite diagnoses were mainly rheumatoid arthritis (9%) and septic arthritis (5%). Some previous studies on patients with undifferentiated chronic monoarthritis have revealed rheumatoid arthritis and spondyloarthropathy as the most frequent diagnoses [8, 16]. Another study on patients with chronic monoarthritis reported RA in 9% and crystal arthropathy in 7.4% of cases [15]. Consistent with our findings, RA ranks as the most common definite diagnosis in cases with a previous diagnosis of undifferentiated chronic monoarthritis. The histopathologic features of non-specific synovitis and rheumatoid arthritis are very similar [11, 17]. Our study demonstrated a higher prevalence of neovascularization, cellular infiltration, synovial and endothelial proliferation, pannus formation, and fibrosis in patients with non-specific synovitis than the ones with a definite histopathologic diagnosis. Among the findings of earlier stages of RA are synovial infiltration, endothelial proliferation, high vascularity, and fibrin deposition. Most of these features are found in the early stages of rheumatoid arthritis [17–19] and other types of synovitis as well [20]. Based on what was mentioned, it can be deduced that RA begins with non-specific synovitis and gradually transits into established RA. The diagnostic challenge in distinguishing early phases of RA from non-specific synovitis is common. Repeated serial biopsies may confirm the diagnosis of RA in most cases with non-specific synovitis, although its invasiveness limits its use in clinical practice. Using molecular and immunohistochemistry findings might help differentiate these two entities [7].

The second most common diagnosis in the present study was septic arthritis. The diagnosis of septic arthritis was confirmed with a positive culture of the samples taken from the patients or evidence of infection observed by the pathologist. Given the negative culture in some cases of septic arthritis, some other factors may aid in making the diagnosis and preventing joint destruction. Synovial white blood cell count [21], neutrophil count [21, 22], and the neutrophil-to-lymphocyte ratio [23] are among the suggested surrogate diagnostic markers for septic arthritis. The onset of septic arthritis is followed by chemo-attraction of neutrophils as the main immune cells combating the infection. Neutrophils act against the spread of infection through the entrapment of microorganisms with neutrophil extracellular traps, phagocytosis of the microorganisms, and recruitment of additional immune cells to the infected joint. Based on previous studies, the increased neutrophil count predicts joint infection and damage [22]. Given the low sensitivity of synovial white blood cells and neutrophil count (56% and 60–65%), the neutrophil-to-lymphocyte ratio can be recruited as a sensitive surrogate marker of diagnosis. A previous study found 78% sensitivity and 81% specificity for synovial neutrophil-to-lymphocyte ratio larger than 25 and recommended this marker to be considered in clinical decision-making [23]. The present study adds to the present literature regarding the similarities of undifferentiated monoarthritis and rheumatoid arthritis and emphasizes the follow-up of these patients. The most important limitations of our study were the small sample size, the study’s retrospective nature, and the lack of patient follow-up. Another limitation of the present study was the blind biopsy that restricted the targeted sampling of the synovium. Arthroscopy is a recently-introduced modality that allows macroscopic assessment of the synovium and aids in diagnosis and targeted biopsy [24]. Recent studies demonstrate the superiority of ultrasound and arthroscopy in guiding the biopsies and yielding better synovial samples. The mentioned superiority is specifically evident in biopsies taken from large joints, including the knees [25]. Thus, we suggest assessing the findings with biopsies taken with either arthroscopy or the guide of ultrasound. Another limitation was the lack of genetic, molecular, and immunohistochemistry evaluation of the samples. Evaluating these variables in future studies can optimize the utility of synovial biopsy for making a definite diagnosis of chronic knee monoarthritis.

## Conclusion

Although synovial biopsy can elucidate the diagnosis in about one-third of the cases with undifferentiated chronic knee monoarthritis, non-specific synovitis remains the most common pathologic label in evaluating chronic knee monoarthritis. As a result, the main decision-making for the diagnosis in the remaining two-thirds of the monoarthritis patients is based on the physician’s expert opinion and the results of the patients’ future follow-ups.

## Data Availability

The dataset used and analyzed during the current study is available from the corresponding author on reasonable request.

## Abbreviations

CPPD: Calcium pyrophosphate crystal deposition disease
RA: Rheumatoid arthritis
CBC: Complete blood count
ESR: Erythrocyte sedimentation rate
CRP: C-Reactive protein
ANA: antinuclear antibody
TSH: Thyroid stimulating hormone
APCAs: Anti-cyclic citrullinated peptides antibodies
